# Selenium-Biofortified Probiotics: A Synergistic Microbial–Nutritional Strategy Against Exercise-Induced Stress

**DOI:** 10.3390/nu18060958

**Published:** 2026-03-18

**Authors:** Qi Wang, Jinjin Xing, Yujing Huang, Jiaqiang Huang, Kongdi Zhu, Xia Zhang

**Affiliations:** 1 Institute of Artificial Intelligence in Sports, Capital University of Physical Education and Sports, Beijing 100083, China; 2 Institute of Agricultural Product Development and Food Science, Xizang Academy of Agricultural and Animal Husbandry Sciences, Lhasa 850000, China; 3 Key Laboratory of Precision Nutrition and Food Quality, Ministry of Education, Department of Nutrition and Health, China Agricultural University, Beijing 100083, China

**Keywords:** selenium, probiotics, sports nutrition, oxidative stress, gut barrier, gut–muscle axis, synergistic effect, athletic performance

## Abstract

This review aims to explore the potential and mechanisms of selenium-biofortified probiotics as an innovative nutritional strategy for alleviating exercise-induced physiological stress. Exercise, particularly high-intensity or exhaustive exercise, triggers a cascade of physiological perturbations, including oxidative stress, inflammatory responses, gut barrier dysfunction, and muscle damage. Traditional single-nutrient strategies, such as inorganic selenium or probiotic supplementation, are often limited by low bioavailability or a narrow scope of action. Selenium-biofortified probiotics are produced via microbial biotransformation, which converts inorganic selenium into bioavailable organic forms like selenoamino acids or selenium nanoparticles that are loaded onto active probiotic carriers. This creates a synergistic entity combining the bioactivity of selenium with the gut-modulating functions of probiotics. Their core mechanism involves establishing a multi-layered defense system: by providing substrate for key selenoproteins like glutathione peroxidase, they directly enhance endogenous antioxidant defenses; by modulating immune cytokine networks, they downregulate excessive post-exercise inflammation; through probiotic colonization and metabolites, they maintain intestinal epithelial barrier integrity, countering exercise-induced intestinal hyperpermeability; and via the gut–muscle axis, they may regulate muscle metabolism and repair. Animal studies provide evidence for improved exercise endurance and reduced damage markers, but human clinical trials show inconsistent results, highlighting the influence of study design, dosage, and individual baseline status. Future research requires high-quality, long-term human trials to elucidate specific molecular pathways and develop personalized application protocols, advancing this synergistic strategy toward precision sports nutrition.

## 1. Introduction

Regular exercise is a cornerstone of health promotion. However, acute, high-intensity, or exhaustive exercise inevitably imposes significant physiological stress, manifesting as an interconnected cascade of core disturbances [1]. The dramatic increase in energy metabolism during exercise leads to excessive generation of reactive oxygen species (ROS). When this production surpasses the clearance capacity of endogenous antioxidant systems, oxidative stress ensues, causing damage to lipids, proteins, and DNA [2,3]. Concurrently, oxidative stress and tissue micro-damage trigger a sterile inflammatory response, elevating levels of pro-inflammatory cytokines such as TNF-α and IL-6 [4]. Furthermore, intense exercise can induce gastrointestinal ischemia–reperfusion injury, disrupting intestinal epithelial tight junctions and increasing permeability—a condition termed exercise-induced intestinal hyperpermeability. This allows endotoxin translocation into the bloodstream, provoking systemic inflammation [5]. This interconnected triad of oxidative stress, inflammation, and gut barrier disruption culminates in accelerated fatigue, impaired muscle function, delayed recovery, and increased susceptibility to overtraining and illness [6].

Confronting this complex challenge necessitates moving beyond traditional single-target nutritional interventions. The trace element selenium is of fundamental importance, not merely as a generic antioxidant, but as an essential component of the selenoprotein family. In humans, 25 selenoproteins have been identified, and they function as the primary executors of selenium’s biological roles [7,8,9]. These proteins are critical for maintaining cellular and tissue homeostasis, with functions spanning thyroid hormone metabolism, intracellular and extracellular antioxidation, and protein quality control within the endoplasmic reticulum [10,11]. In the context of skeletal muscle—the primary organ for locomotion and a major site of exercise-induced stress—selenoproteins are indispensable. Emerging evidence highlights their critical role in reducing exercise-induced fatigue, improving post-exercise recovery, and combating age-related muscle decline [12]. Selenium deficiency disrupts this system, impairing maximal contractile strength, causing postural instability, and contributing to myopathic pathologies and sarcopenia [13,14,15]. Therefore, strategies aimed at optimizing selenium status hold significant promise in sports nutrition.

However, isolated strategies often fail to address the multidimensional nature of exercise stress cohesively. For instance, supplementing with the trace element selenium, an essential component of antioxidant enzymes like glutathione peroxidase, aims to bolster antioxidant defense [1], but such inorganic selenium forms often exhibit low bioavailability and a narrow therapeutic index [9,16]. Importantly, evidence from poultry nutrition research indicates that nano-selenium exhibits superior bioavailability and more efficient selenoprotein synthesis compared to inorganic and organic forms, which may translate to enhanced efficacy of SeNP-fortified probiotics. Furthermore, selenium plays crucial roles in mitigating various stressors, including heat stress and toxin exposure, primarily through antioxidant and anti-inflammatory pathways involving selenoprotein regulation [17,18,19,20]. Conversely, probiotic supplementation aims to stabilize the gut ecosystem, enhance barrier function, and modulate immunity [21]. These isolated strategies fail to address the multidimensional nature of exercise stress cohesively. Therefore, sports nutrition requires an innovative paradigm that employs a systemic and synergistic approach.

Selenium-biofortified probiotics emerge as a compelling integrative strategy. This approach utilizes the biotransformation capability of specific probiotic strains, such as Lactobacillus rhamnosus, to synthesize and load inorganic selenium in situ into more biocompatible and bioavailable organic forms [22]. During cultivation in selenium-supplemented media, these strains incorporate selenium into cellular proteins and/or reduce it to elemental, nano-sized selenium (SeNPs) [23,24]. The resultant product is a functional complex wherein a bioavailable organic selenium source is delivered by a viable probiotic carrier, synergizing the antioxidative and immunomodulatory properties of selenium with the gut-stabilizing and immunoregulatory effects of probiotics [25,26,27]. This review will systematically elaborate on the scientific foundation of selenium-biofortified probiotics, detail their synergistic mechanisms of action, critically evaluate evidence from animal and human studies, and discuss future directions for their application in precision sports nutrition. While the mechanistic framework presented here is primarily derived from foundational research and preclinical models, it is crucial to acknowledge from the outset that the translation of these promising findings into human sports nutrition practice is still in its early stages. As will be critically examined later in this review, the efficacy of selenium-biofortified probiotics, like other selenium interventions, is highly context-dependent and may not directly extrapolate from animal studies to well-nourished athlete populations. Therefore, this review will not only explore the synergistic potential but also rigorously evaluate the existing evidence base, highlighting the critical gaps and controversies that must be addressed to move the field forward.

## 2. Scientific Basis: From Production to Bioactivity

The production of selenium-biofortified probiotics centers on harnessing microbial metabolism for selenium speciation and loading. A common method involves culturing probiotics like lactobacilli or yeast in media containing selenite. The microbial cells reduce and biomineralize the inorganic selenium (Se(IV)) into less toxic, elemental zero-valent SeNPs, which accumulate intracellularly or are secreted [23,28]. The biosynthesis of selenium-biofortified probiotics relies on microbial selenium metabolism. Tolerant strains of Lactobacillus, Bifidobacterium, and other genera, when cultured in media supplemented with sodium selenite, actively assimilate selenium. Intracellularly, selenite (Se^4+^) is reduced, leading to its specific incorporation into selenoproteins via selenocysteine or non-specific integration into general proteins as selenomethionine. Concurrently, metabolic reduction can yield insoluble, elemental selenium (Se^0^) that accumulates as intracellular or extracellular nanoparticles [29]. These biosynthesized nanoparticles typically demonstrate uniform size, good stability, and high biocompatibility. Furthermore, selenium can replace sulfur in sulfur-containing amino acids, integrating into microbial proteins as selenomethionine (SeMet) or selenocysteine (Sec) to form a natural organic selenium reservoir [30]. Selenium-enriched yeast is a classic functional food ingredient produced via this route. Strain selection is paramount, as efficiency of selenium uptake, tolerance thresholds, and the resultant speciation profile vary considerably [31]. Certain Lactobacillus plantarum strains demonstrate high selenium accumulation capacity and resilience [32].

### 2.1. The Advantage of Organic and Nano Forms

Consequently, selenium-biofortified probiotics possess a dual bioactive identity. Primarily, they serve as an efficient delivery vehicle for organic selenium. Comparative pharmacokinetic studies in rodents indicate that selenium from enriched probiotics is absorbed more efficiently and exhibits longer plasma retention than inorganic selenite, leading to superior tissue selenium accretion and more potent upregulation of selenoenzyme activities like GPx [16]. This aligns with broader evidence that organic Se (such as selenomethionine and Se yeast) and nano-selenium (SeNPs) have better bioavailability than inorganic Se [16,33,34]. Beyond the fundamental improvement in bioavailability, the microbial biotransformation process integral to selenium-biofortified probiotics often leads to the synthesis of selenium nanoparticles (SeNPs) [26,35]. These biogenic SeNPs are not merely an alternative selenium form but possess distinct bioactive advantages. They exhibit enhanced free radical scavenging capacity due to their high surface-area-to-volume ratio and can be stabilized by a natural coating of proteins or polysaccharides derived from the probiotic matrix, which prevents aggregation and may facilitate targeted delivery [27,36,37]. Compared to inorganic selenium supplements, the microbially synthesized organic selenium or selenium nanoparticles often show enhanced stability and sustained-release properties in the gastrointestinal tract, leading to more effective elevation of tissue selenium status and antioxidant enzyme activity while potentially mitigating risks associated with hepatic selenium overload [23,34]. Secondly, these constructs retain the inherent benefits of probiotics, including tolerance to gastric acid and bile salts, adhesion to intestinal epithelium, modulation of local and systemic immunity, and production of beneficial metabolites like short-chain fatty acids [38]. Notably, the selenium enrichment process itself can augment certain probiotic traits; selected strains demonstrate enhanced in vitro antioxidant capacity and improved bile salt tolerance following selenium assimilation [39].

### 2.2. The Synergy Rationale in Exercise Physiology

The promise of a synergistic effect stems from the potential for selenium and probiotics to complement and amplify each other’s actions across the various physiological challenges posed by exercise. The rationale for combining these agents is reinforced by extensive research on their individual roles in exercise physiology. Probiotic supplementation has been shown to improve gut barrier function, reducing gastrointestinal symptoms and systemic endotoxin exposure after exhaustive exercise [40,41]. Concurrently, probiotics can modulate post-exercise immune profiles, increasing anti-inflammatory IL-10 and decreasing pro-inflammatory IL-6 in some athlete cohorts [42]. On the other hand, while selenium is crucial for antioxidant defense, its standalone ergogenic effect in well-nourished athletes is questionable [1]. This juxtaposition underscores the logic of a combined approach. Utilizing probiotics to target the gut-based origins of inflammation and permeability, while simultaneously using a bioavailable form of selenium to directly strengthen systemic antioxidant defenses, thereby addressing complementary nodes of the exercise–stress network.

Microbe-mediated biotransformation is a crucial pathway for producing efficient and safe organic selenium supplements. Future research needs to optimize strain selection and fermentation processes to obtain engineered probiotic strains with specific functionalities (e.g., high-efficiency SeNP synthesis, strong gut colonization).

## 3. Core Mechanisms: Constructing a Multi-Layered Synergistic Defense System

### 3.1. Proposed Synergistic Mechanisms of Action

**Figure 1:**
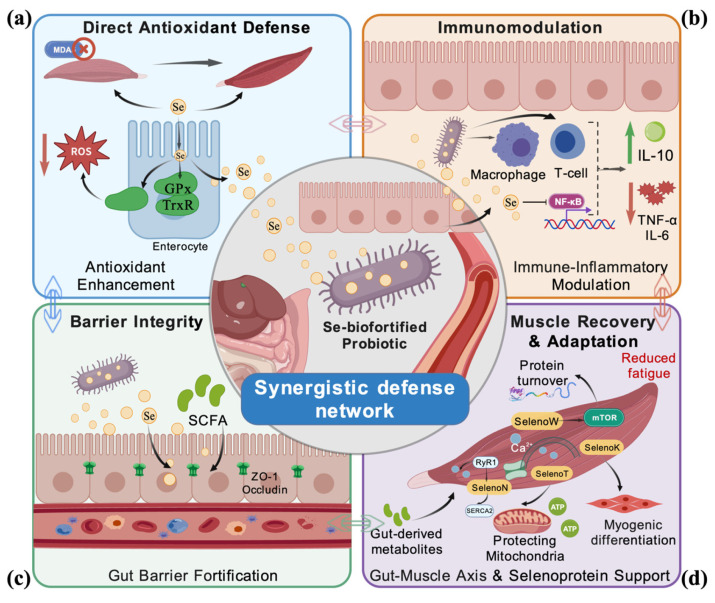
Schematic diagram of the core synergistic mechanisms by which selenium-biofortified probiotics alleviate exercise-induced stress. The figure illustrates four interconnected layers of defense: (**a**) Antioxidant enhancement: Probiotic-delivered selenium supports host synthesis of selenoproteins, including glutathione peroxidase (GPx) and thioredoxin reductase (TrxR). (**b**) Immune-inflammatory modulation: Selenium and probiotic signals downregulate pro-inflammatory cytokine production (e.g., interleukin-6, IL-6; tumor necrosis factor-alpha, TNF-α) via modulation of the nuclear factor kappa-light-chain-enhancer of activated B cells (NF-κB) pathway, while upregulating anti-inflammatory cytokines such as interleukin-10 (IL-10). (**c**) Gut barrier fortification: Probiotics enhance tight junction integrity through proteins including occludin and zonula occludens-1 (ZO-1), and produce protective metabolites such as short-chain fatty acids (SCFAs). (**d**) Gut–muscle axis influence: Probiotic-modulated microbiota generate systemic metabolites that may influence skeletal muscle metabolism and function. Created with BioGDP.com.

The distinctive value of selenium-biofortified probiotics lies in their capacity to engage multiple key pathways of exercise stress, thereby constructing an integrated, synergistic defense network, as illustrated in Figure 1.

### 3.2. Direct Enhancement of Endogenous Antioxidant Defenses

Selenium is incorporated as selenocysteine into the active sites of critical antioxidant enzymes, including glutathione peroxidase (GPx) and thioredoxin reductase (TrxR). Post-exercise supplementation aims to supply adequate substrate for the synthesis of these enzymes, thereby boosting the body’s capacity to neutralize peroxides. Research indicates that organic selenium supplementation can significantly increase GPx activity in plasma, erythrocytes, and muscle tissue post-exercise while reducing biomarkers of lipid peroxidation, such as malondialdehyde (MDA) [1,3]. The antioxidant prowess of selenium is primarily mediated by selenoproteins. The GPx family serves as a pivotal regulator of ROS. While GPx1 scavenges cytosolic hydrogen peroxide, the mitochondrial isoform GPx4 plays a unique and critical role in preventing ferroptosis by reducing lipid hydroperoxides within mitochondrial membranes, thus safeguarding electron transport chain integrity during intense exercise [43,44]. In endurance athletes, muscle GPx activity can increase significantly post-training, protecting mitochondrial membranes from oxidative rupture [45]. Another key system is the thioredoxin reductase (Txnrd) family, which reduces oxidized thioredoxin (Trx), maintaining the intracellular reducing environment essential for protein function [46]. Methionine sulfoxide reductase B1 (MsrB1), another selenoprotein, protects proteins from oxidative damage and may be involved in actin dynamics relevant to muscle function [47,48].

Animal studies confirm that selenium from enriched probiotics elevates tissue GPx activity and reduces oxidative damage markers like MDA [49]. In human exercise models, selenium supplementation mitigates exercise-induced rises in lipid peroxidation [50]. Selenium-biofortified probiotics may offer an advantage by providing selenium in a form with potentially superior bioavailability, leading to more efficient upregulation of critical selenoprotein activity. Supporting this, selenium nanoparticles synthesized by Lactobacillus rhamnosus maintained potent antioxidant capacity even under simulated digestive conditions [23].

### 3.3. Modulation of Immune and Inflammatory Balance

Intense exercise often induces a transient state of immune perturbation and elevated inflammation. Both selenium and probiotics exhibit immunomodulatory properties. Selenium deficiency can exacerbate inflammatory responses, whereas adequate selenium status helps modulate pathways like NF-κB to reduce the production of pro-inflammatory cytokines [34,51]. Specific selenoproteins like SELENOS (Selenoprotein S) have been identified as having protective and anti-inflammatory effects in vivo, and its polymorphisms are linked to inflammatory dysregulation [52,53]. Probiotics modulate gut immune cell function, promoting the secretion of anti-inflammatory cytokines like IL-10. Their combination may yield additive or synergistic effects. In tumor model studies, exercise combined with SeNPs supplementation reduced IL-4 levels in tumor tissue and increased Th1-type cytokines in the spleen, suggesting a positive modulation of immune response direction [4]. In the exercise context, such combined regulation holds promise for suppressing excessive inflammation while maintaining normal immune surveillance.

We have found some cross-species evidence regarding Selenium’s Anti-inflammatory Pathways. Research in poultry immunology elucidates specific molecular pathways through which selenium modulates inflammation. Selenium deficiency in chickens leads to splenic damage characterized by the inhibition of the IGF-1R/PI3K/Akt/mTOR pathway and activation of the DUSP1/NF-κB pathway, promoting inflammation and apoptosis [54,55]. Supplemental selenium, on the other hand, antagonizes these effects. For example, selenium mitigates heavy metal (e.g., cadmium, lead) induced immunotoxicity by activating the PI3K/Akt pathway and inhibiting the MAPK/NF-κB pathway in immune organs, reducing pro-inflammatory cytokine production and cell death [56,57]. These conserved pathways (PI3K/Akt, NF-κB, MAPK) are also central to exercise-induced inflammation. Therefore, selenium delivered via probiotics could similarly dampen post-exercise inflammatory signaling through these well-established mechanisms. While these findings from poultry models illustrate conserved pathways (PI3K/Akt, NF-κB, MAPK) that are also central to exercise-induced inflammation in humans, direct confirmation in human athletes is required.

### 3.4. Maintenance of Intestinal Barrier Integrity

The compromise of intestinal barrier function during strenuous exercise is a key contributor to systemic inflammation. A principal function of probiotics is to preserve and restore gut barrier integrity. They achieve this by competitively excluding pathogens, stimulating the expression of tight junction proteins (Occludin, ZO-1), and enhancing mucus production [5]. Selenium may also play a supportive role via selenoproteins localized in the endoplasmic reticulum (ER), such as SELENOF, SELENOK, SELENOS, SELENOM, and SELENOT, which are involved in managing ER stress—a contributor to barrier dysfunction [58,59]. When probiotics are fortified with selenium, their tolerance to oxidative stress may be enhanced, allowing them to survive better in the oxidative intestinal environment induced by exercise and execute barrier-protective functions more effectively. Probiotics support barrier integrity by enhancing tight junction protein expression and producing barrier-strengthening metabolites like butyrate. The integrated selenium may further bolster this role by enhancing the probiotic bacteria’s own oxidative stress resilience [39]. Although direct literature on selenium-biofortified probiotics and exercise-induced leaky gut is limited, the established gut-protective roles of probiotics and the observed systemic anti-inflammatory effects of selenium-biofortified complexes strongly support this mechanistic avenue. While these findings are derived primarily from animal models and in vitro studies, they provide a mechanistic rationale for the potential gut-protective effects of selenium-biofortified probiotics in humans. Direct validation in athletic populations undergoing exercise-induced intestinal stress is needed.

### 3.5. Regulation of the Gut–Muscle Axis to Promote Recovery

The gut–muscle axis is an emerging frontier in exercise science, referring to the remote regulation of skeletal muscle metabolism, inflammation, and repair by gut microbiota and their metabolites such as short-chain fatty acids (SCFAs) via immune, neural, and endocrine pathways [60]. Exercise can alter gut microbiota composition, and specific probiotic supplementation can reverse detrimental exercise-induced microbial shifts. Selenium, as a trace element for microbial growth, may also influence microbiota structure [61]. Selenium-biofortified probiotics may thus dually shape a healthier gut microbiome, leading to increased production of metabolites like short-chain fatty acids like butyrate. These metabolites can enter systemic circulation and exert direct effects on muscle, such as modulating protein turnover, promoting muscle protein synthesis, inhibiting catabolism, reducing muscle inflammation, and influencing energy metabolism. For instance, exercise combined with supplementation increased the expression of factors like Irisin and Sema3A in muscle and serum, which are linked to energy metabolism and neuroprotection, hinting at systemic metabolic crosstalk [62]. Emerging evidence links microbiota to muscle physiology. For instance, selenium supplementation alleviated muscle atrophy in mice, an effect correlated with beneficial shifts in gut microbiota [63]. By promoting a healthier gut microbiota profile, these probiotics could foster a systemic environment conducive to muscle repair and recovery post-exercise. Investigating whether and how selenium-biofortified probiotics influence post-exercise recovery via the gut–muscle axis is a promising research direction. Although these animal studies establish a plausible gut–muscle axis and suggest beneficial effects of selenium and probiotics on muscle recovery, human data remain sparse. Whether similar mechanisms operate in athletes and translate to improved performance or recovery requires direct investigation.

### 3.6. Support of Muscle-Specific Selenoproteins

Beyond gut-derived signals, the bioavailable selenium delivered by these probiotics is crucial for the synthesis of skeletal muscle-specific selenoproteins that are directly involved in adaptation and repair [64]. Selenoprotein W (SELENOW) modulates protein turnover via the RAC1-mTOR axis, coordinating protein synthesis and degradation. Its knockout exacerbates muscle loss by suppressing mTOR signaling and activating ubiquitin ligases (MuRF1/Atrogin-1) [65]. Its expression is positively correlated with grip strength in the elderly [15]. Selenoprotein K (SELENOK) is required for satellite cell-mediated myogenic differentiation and protects skeletal muscle from damage [66]. SELENOW also plays a pivotal role in myogenic differentiation and myoblast fusion [67,68]. Selenoprotein N (SELENON) regulates ER calcium levels by interacting with ryanodine receptors (RyR1) and the SERCA2 pump. Its deficiency leads to calcium handling defects, contributing to muscle fatigue and congenital myopathies [69,70,71]. SELENOT is also involved in regulating intracellular calcium concentration, affecting factors like STIM1 and TRPC1 that are crucial for myogenesis; Selenoprotein T (SELENOT) protects against mitochondrial oxidative stress, maintains ATP production, and promotes myoblast proliferation [72,73]. Other selenoproteins like SELENOH and SELENOO also contribute to mitochondrial biogenesis and redox function [74,75]. By promoting a healthier gut environment and simultaneously providing the substrate for these critical selenoproteins, selenium-biofortified probiotics could foster a systemic and cellular environment highly conducive to post-exercise muscle repair, adaptation, and recovery. Collectively, these muscle-specific selenoproteins are not merely passive antioxidants but active regulators of key processes governing muscle adaptation to exercise: protein turnover (SELENOW), regeneration (SELENOK), calcium handling and contraction (SELENON), and mitochondrial energy production (SELENOT). By providing the essential substrate for their synthesis, selenium-biofortified probiotics have the potential to directly support these fundamental aspects of muscle health and performance at the cellular level.

### 3.7. Potential for Stress Resistance: Insights from Non-Exercise Stressors

The ability of selenium to enhance systemic resilience under stress is not limited to exercise. In poultry, selenium plays a pivotal role in mitigating heat stress—a physiologically relevant model for hyperthermia induced by intense exercise. Selenium supplementation (as sodium selenite, selenium yeast, selenomethionine, or nanoselenium) alleviates heat stress-induced disorders in hepatic lipid metabolism, mitochondrial dysfunction, and endoplasmic reticulum stress (ERS) in broilers [76,77]. The mechanisms involve upregulation of key hepatic selenoproteins and modulation of the AMPK signaling pathway. Furthermore, selenium effectively detoxifies various stressors like heavy metals (Cd, Pb, Hg) and mycotoxins (e.g., aflatoxin B1) in poultry, primarily by attenuating oxidative stress, inflammatory responses, and specific cell death pathways (apoptosis, necroptosis, ferroptosis), often through the regulation of selenoproteins and signaling pathways like Nrf2 and PI3K/Akt [78,79,80]. This broad-spectrum stress-protective role of selenium, mediated through conserved redox and inflammatory pathways, strongly supports its potential as a core component in a probiotic-based strategy designed to enhance multi-system resilience during and after exhaustive exercise. These observations from poultry models highlight conserved stress-response pathways that may also be relevant to exercise-induced stress in humans. However, extrapolation must be cautious, and human studies are essential to confirm whether these protective effects extend to athletes under high-intensity training.

This multi-target mechanism is supported by patterns observed in separate lines of research. For instance, the administration of Lactobacillus plantarum PL-02 combined with resistance training in mice not only improved exercise performance but also significantly reduced post-exercise lactate, ammonia, and creatine kinase levels, indicating a combined effect on energy metabolism, fatigue, and muscle damage [81]. Similarly, other probiotic strains have been shown to alleviate oxidative stress parameters, such as improving thiol/disulfide homeostasis in exercising rats [82]. These effects mirror the desired outcomes of selenium supplementation but are achieved through gut-mediated pathways. The integration of selenium directly into such a probiotic vector could therefore theoretically amplify these benefits by adding a direct, systemic antioxidant effector mechanism to the gut-initiated protective cascades. The action of selenium-biofortified probiotics is systemic. Future mechanistic studies should employ multi-omics (microbiome, metabolome, transcriptome) integration to panoramically dissect their cross-organ signaling networks from the gut to the muscle.

## 4. Critical Translational Analysis from Preclinical Models to Human Studies

The translation of basic selenium (Se) research into practical nutritional applications, particularly within the context of physical performance and stress resilience, requires a critical examination of evidence across the research continuum. This section synthesizes and evaluates findings from animal models and human trials, highlighting both the demonstrated potential and the significant translational challenges associated with Se supplementation, with a specific focus on emerging formats like Se-biofortified probiotics.

### 4.1. Positive Evidence from Animal Model Studies

Animal studies provide robust proof-of-concept, consistently supporting the beneficial role of Se supplementation in models of physiological stress and exercise. Supplementation with inorganic Se, organic Se species, or selenium nanoparticles (SeNPs) has been shown to enhance endurance capacity, as evidenced by prolonged time to exhaustion in forced swim tests [83,84]. These interventions also attenuate exercise-induced tissue damage, reflected in reduced post-exercise serum levels of muscle damage markers such as creatine kinase (CK), lactate dehydrogenase (LDH), and blood urea nitrogen (BUN) [85,86]. Further benefits include the optimization of energy metabolism through increased glycogen storage [83] and protection against combined stressors, such as exercise coupled with cigarette smoke extract, mitigating damage to lung tissue and mitochondrial function [62,87]. Specific selenoprotein-focused studies in animal models reveal that selenium deficiency leads to mitochondrial ROS overproduction, suppressed myoblast proliferation, and increased apoptosis, which can be rescued by selenium supplementation or specific selenoproteins like SELENOT [73], Furthermore, selenium supplementation has been shown to alleviate liver injury induced by exercise fatigue and ameliorate oxidative, energetic, metabolic, and endocrine imbalances in rat skeletal muscle [88].

Notably, a compelling body of preclinical research directly investigates Se-biofortified probiotics, suggesting superior or synergistic efficacy compared to conventional Se supplements. In murine models of metabolic stress (e.g., high-fat diet), Se-enriched probiotics were more effective than equivalent doses of sodium selenite or non-fortified probiotics in improving systemic lipid profiles, enhancing hepatic antioxidant enzyme activities (e.g., superoxide dismutase (SOD) and glutathione peroxidase (GSH-Px)), and reducing histopathological liver damage [89]. In a rat model of heat stress, supplementation with Se-enriched probiotics effectively attenuated liver injury (normalizing serum AST and ALT), boosted systemic antioxidant capacity, and significantly downregulated hepatic expression of key pro-inflammatory mediators, including IL-6, TNF-α, and NF-κB [51]. Earlier foundational work also indicates that Se-enriched probiotics can enhance host immune parameters, such as macrophage phagocytic activity, more effectively than inorganic Se sources [90]. This collective evidence underscores that the benefits extend beyond mere Se delivery, implying a synergistic effect where the probiotic carrier may enhance bioavailability, provide concurrent gut health benefits, or modulate the host’s response to Se.

Advanced delivery systems further refine this approach. For instance, SeNPs synthesized by Lactobacillus rhamnosus and subsequently loaded onto Ganoderma lucidum spores demonstrated higher in vitro antioxidant activity, superior sustained-release properties in simulated gastrointestinal fluids, and more effectively alleviated fatigue indicators in a mouse exercise model. Notably, this complex resulted in lower hepatic Se accumulation compared to isolated SeNPs, highlighting a promising profile for efficient delivery with potentially reduced risk of tissue overload.

This body of evidence underscores that the benefits extend beyond the provision of selenium alone, suggesting a synergistic effect conferred by the probiotic delivery system. Pioneering formulation work has further refined this approach. For instance, SeNPs synthesized by Lactobacillus rhamnosus and subsequently loaded onto Ganoderma lucidum spores demonstrated higher in vitro antioxidant activity, superior sustained-release properties in simulated gastrointestinal fluids, and more effectively alleviated fatigue indicators in a mouse exercise model. Notably, this complex resulted in lower hepatic Se accumulation compared to isolated SeNPs, highlighting a promising profile for efficient delivery with potentially reduced risk of tissue overload [23].

### 4.2. Controversies and Limitations in Human Clinical Trials

The robust and reproducible benefits observed in preclinical models contrast sharply with the inconsistent and often null findings reported from human studies, particularly in athletic populations. This translational gap represents a fundamental hurdle in advancing selenium-biofortified probiotics from a mechanistically appealing concept to a clinically validated sports nutrition strategy. It compels a critical re-evaluation: efficacy is not an intrinsic attribute of a supplement, but rather a context-dependent outcome shaped by the interplay between the intervention and the recipient’s physiological status, most critically, baseline selenium and antioxidant levels (Table 1). The studies compiled in Table 1 illustrate this principle: selenium supplementation consistently improves outcomes in individuals with suboptimal status or heightened oxidative stress, yet confers no detectable benefit in replete individuals, reflecting the principle of diminishing returns that governs trace element nutrition.

Understanding the sources of this heterogeneity requires a systematic examination of how differences in study design, participant characteristics, and outcome metrics influence the apparent effectiveness of selenium interventions. Such scrutiny is essential for designing future trials capable of resolving current ambiguities and for developing targeted, evidence-based applications of selenium-biofortified probiotics in sports medicine. Some studies report positive effects. For example, sodium selenite supplementation (200 µg/day for 3 weeks) reduced post-exercise blood levels of lipid hydroperoxides in overweight adults—a population with potentially higher baseline oxidative stress [50]. Combined supplementation with selenium (as sodium selenite, 17.5 µg/day) and vitamin E (400 IU/day) for 3 weeks in small human trials significantly improved antioxidant enzyme activities (SOD, GPx), reduced lipid peroxidation (MDA), and in some cases improved cardiopulmonary endurance (VO_2_max, anaerobic threshold) [91,92,93,94].

However, more high-quality systematic reviews and clinical trials indicate that for athletes with adequate nutritional status, routine selenium supplementation does not significantly enhance aerobic or anaerobic exercise performance [1,95]. An analysis of the UK Biobank cohort even found no significant association between selenium supplement use and fluid intelligence scores, suggesting limited benefits for cognitive function in healthy populations [96]. Equine studies mirror these findings, showing that once dietary selenium requirements are met, additional supplementation offers no further improvement to exercise-induced adaptations or recovery metrics. This underscores the principle of diminishing returns in well-nourished populations [97].

A fundamental distinction must be made between biochemical efficacy and ergogenic effect. A rigorous systematic review concluded that while selenium supplementation (as selenomethionine or selenite) in athletes reliably increases erythrocyte GPx activity and reduces markers of oxidative damage, it confers no measurable enhancement to aerobic or anaerobic exercise performance, strength, or anabolic hormone responses in individuals who are already nutritionally replete [1]. This highlights that correcting a subclinical biochemical deficit is not synonymous with eliciting a super-physiological performance boost.

The dependence on baseline status is vividly illustrated in studies stratified by metabolic health. For instance, sodium selenite supplementation significantly reduced post-exercise lipid peroxide levels in overweight adults, a population with likely higher baseline oxidative stress, but showed no such effect in their normal-weight counterparts [50]. This indicates that the benefit of supplementation is not universal but is targeted toward overcoming a specific physiological limitation.

This concept aligns with the principle of diminishing returns, which is clearly demonstrated in animal athletic models. Research in young athletic horses showed that a standard exercise training program effectively improved antioxidant defenses and reduced post-exercise muscle damage. However, providing dietary selenium at three times the established nutritional requirement offered no additional adaptive advantage over merely meeting the adequate intake level [97]. Once sufficiency is achieved, additional selenium intake appears to yield no further performance or recovery benefit.

Collectively, these findings from conventional selenium supplementation underline a critical evidence gap in the field. There is a pronounced lack of high-quality, randomized, placebo-controlled trials specifically designed to evaluate selenium-biofortified probiotics in human athletic populations. The potential synergistic advantages hypothesized from preclinical studies, such as enhanced bioavailability, gut-mediated immunomodulation, and improved barrier function, remain to be rigorously tested in the complex and variable human exercise context. Future research must therefore not only employ robust designs but also prioritize participant stratification based on baseline selenium status, microbiome profiles, and training loads to identify which athlete subgroups might truly benefit from this integrated nutritional strategy.

**Table 1:** Key Human and Equine Studies on Selenium Supplementation and Exercise Outcomes.

Study (Year)	Subjects	Intervention	Exercise Protocol	Key Findings	Limitations/Implications
Fernandez-Lazaro et al. (2020) [1]	Athletes/Active Individuals	Selenomethionine (180–240 µg/day) or Sodium Selenite (200 µg/day)	Various training	Significantly increased GPx activity, decreased lipid peroxides; did not improve exercise performance, testosterone levels, or training adaptations.	Confirms antioxidant role but negates ergogenic effect in replete individuals.
Savory et al. (2012) [50]	Overweight & Normal-weight Adults	Sodium Selenite (200 µg/day) or placebo, 3 weeks	Cycling at 70% VO_2_max for 30 min	Reduced oxidative stress only in the overweight group post-exercise.	Efficacy is population-specific, dependent on baseline oxidative stress.
White & Warren (2017) [97]	Young Horses	Dietary Se: 0.1 vs. 0.3 mg/kg DM, 14 weeks	Submaximal exercise training	Training itself improved antioxidant defense and reduced post-exercise muscle damage; extra Se provided no further gain.	Highlights importance of meeting, but not exceeding, nutritional requirements.
Lehrer & Rheinstein (2022) [96]	UK Biobank Cohort	Selenium Supplement Use (Yes/No)	Habitual Physical Activity	No significant association between Se supplementation and fluid intelligence scores; effect dwarfed by age, education, vigorous activity	Questions the benefit of supplements for higher cognitive functions in nutritionally replete populations.
Tessier et al. (1995) [45]	Healthy Males	Organic Selenium (240 µg/day) or placebo, 10 weeks	Endurance training and acute exhaustive exercise	The Se group showed a trend (*p* = 0.057) for increased muscle GPx activity in response to acute exercise after training.	Suggests Se may influence the pattern of enzymatic response to acute stress following training.

These contradictory outcomes likely stem from several factors: (1) Baseline Nutritional Status: The daily selenium intake and plasma selenium levels of participants are crucial determinants of supplementation efficacy. Deficient individuals show clear benefits, while sufficient individuals do not [23,98]. (2) Supplement Form and Dose: The bioavailability and activity of organic selenium (e.g., SeMet, SeNPs) may be superior to inorganic selenium, but the optimal dose for exercise supplementation remains undefined. (3) Exercise Type and Intensity: Different exercise modalities produce distinct oxidative stress and physiological demands, potentially requiring differentiated nutritional strategies. (4) Outcome Measures: Using “exercise performance” as an endpoint may be insufficiently sensitive; greater focus should be placed on recovery rate, damage markers, immune function, and subjective fatigue. Currently, high-quality RCTs specifically investigating “selenium-biofortified probiotics” in athletes are almost entirely lacking, representing the largest gap in the evidence chain.

## 5. Discussion and Future Perspectives: Towards Precision Sports Nutrition

Selenium-biofortified probiotics represent an innovative approach combining trace element nutrition with microbiome intervention. The mechanisms outlined in this review suggest that their potential advantage lies in systemically addressing multiple facets of exercise stress via the gut, a central hub, rather than locally. However, translating this promising concept into widespread sports nutrition practice requires addressing several key issues.

### 5.1. Deepening Mechanistic Understanding

To date, research has largely centered on terminal outcomes, including fatigue relief, endurance gains, and reductions in damage markers, while the intermediate processes driving these effects remain largely uncharted. Several fundamental questions have yet to be systematically addressed. These include how the probiotic carrier shapes the metabolic fate of ingested selenium; whether bacterial biotransformation into selenoproteins or synthesis of SeNPs alters selenium speciation, absorption kinetics, or tissue distribution; and through which molecular signals selenium and probiotics converge along the gut–muscle axis to influence systemic metabolism and muscle function.

Answering these questions will require moving beyond descriptive outcome measures toward mechanistic dissection using integrated experimental platforms. Stable isotope-labeled selenium tracers, combined with speciation analysis, could map the in vivo trajectory of probiotic-delivered selenium, revealing how bacterial metabolism influences host selenium bioavailability [99]. Multi-omics integration, including metagenomics to track probiotic engraftment and microbiome shifts, metabolomics to identify gut-derived bioactive metabolites, and host transcriptomics to profile tissue responses, offers a systems-level view of the gut–muscle communication network [100]. Complementary in vitro models, such as transwell co-cultures of intestinal epithelial cells colonized by selenium-biofortified probiotics and skeletal myocytes, could enable controlled interrogation of candidate signaling mediators under defined conditions. Such mechanistic insight is not merely academic; it is a prerequisite for rational optimization of strain selection, dosing regimens, and targeted applications in athletic populations.

### 5.2. Selenoprotein-Targeted Interventions and Personalized Nutrition

The paradigm of “one-size-fits-all” supplementation is becoming obsolete, particularly for trace elements like selenium, where efficacy is critically dependent on an individual’s pre-existing status and unique physiology. To translate the potential of selenium-biofortified probiotics into consistent benefits, future strategies must adopt a precision nutrition framework. This framework should pivot from population-wide recommendations to interventions guided by functional biomarkers, multi-omics profiling, and sport-specific physiological demands. The following subsections outline three key dimensions for participant stratification, each grounded in the mechanistic discussions presented earlier. It is important to reiterate that these approaches represent a forward-looking, hypothesis-driven framework and require rigorous clinical validation before they can inform evidence-based practice.

#### 5.2.1. Functional Selenium Status

While plasma or serum total selenium concentration provides a basic measure of selenium exposure, it does not necessarily reflect functional selenium status at the tissue level. Selenoprotein P (SELENOP), the primary hepatokine responsible for systemic selenium distribution and delivery to peripheral tissues, represents a superior functional biomarker. Plasma SELENOP concentration correlates more closely with tissue selenoenzyme activity (e.g., GPx activity in erythrocytes, muscle, and other tissues) and with long-term selenium adequacy than does total selenium [101,102]. Moreover, SELENOP responds to changes in selenium intake in a dose-dependent manner and reaches a plateau when selenium requirements are fully met, making it a useful indicator of functional repletion.

In the context of sports nutrition, routine measurement of SELENOP in athletes could serve two critical purposes. First, it can effectively identify individuals with suboptimal functional selenium reserves, i.e., those who are most likely to derive meaningful physiological benefits from supplementation. Second, it can identify selenium-replete individuals for whom additional supplementation would be superfluous or potentially harmful, thereby avoiding unnecessary intake and enabling more efficient resource allocation in research and practice. Incorporating SELENOP as a stratification biomarker in future clinical trials would represent a significant step toward precision in selenium interventions. However, widespread adoption will require validation of SELENOP assays across laboratories, establishment of sport-specific reference ranges, and prospective studies demonstrating that SELENOP-guided supplementation improves clinical outcomes compared to conventional approaches.

#### 5.2.2. Host Genetics and Baseline Microbiome Composition

Genetic variation in pathways governing selenoprotein synthesis and redox homeostasis represents an important determinant of interindividual variability in response to both selenium supplementation and exercise stress. Polymorphisms in selenoprotein-related genes have been associated with differential inflammatory and antioxidant capacity. For instance, variants in SEPS1 (Selenoprotein S) have been linked to altered inflammatory cytokine production and may modulate an individual’s susceptibility to exercise-induced inflammation as well as their responsiveness to selenium’s anti-inflammatory effects [52,53]. Similarly, polymorphisms in glutathione peroxidase genes (GPX1, GPX4) can influence baseline antioxidant enzyme activity and its inducibility by selenium, potentially affecting the magnitude of oxidative defense enhancement achievable through supplementation [103,104]. Beyond these well-studied examples, genes involved in the selenoprotein synthesis machinery (e.g., SECISBP2, TRU-TCA1-1) and those encoding other selenoproteins (e.g., SELENOP, SELENOW) may also harbor functionally relevant variants, though their prevalence and phenotypic consequences in athletic populations remain largely unexplored. As genotyping technologies become increasingly accessible, incorporating targeted genetic profiling into clinical trial designs could help account for the heterogeneity observed in previous studies and enable more precise, genotype-guided supplementation strategies.

In parallel, the composition and functional capacity of an individual’s gut microbiota constitute another critical determinant of response to selenium-biofortified probiotics. The baseline microbiome architecture influences multiple aspects of probiotic efficacy, including strain engraftment, metabolic activity, and the production of bioactive metabolites that may mediate systemic effects via the gut–muscle axis [21]. Metagenomic profiling prior to intervention could serve two complementary purposes. First, it could enable the prediction of an athlete’s compatibility with specific probiotic strains, facilitating rational selection of the most appropriate delivery vehicle for selenium. Second, it could identify baseline microbial signatures associated with favorable responses to selenium-biofortified probiotics, potentially uncovering novel mechanisms of action and informing future strain engineering efforts. The integration of host genetic and microbiome data represents a promising avenue toward multi-dimensional prediction of individual supplementation outcomes, moving beyond univariate stratification toward truly personalized intervention design. Prospective studies that concurrently collect genetic and metagenomic data from adequately powered athlete cohorts are needed to validate this approach and to develop practical algorithms for its implementation in sports nutrition practice.

#### 5.2.3. Sport-Specific Demands

The physiological adaptations elicited by different modes of exercise training are fundamentally distinct, and these differences likely translate into divergent demands on selenium-dependent pathways. A precision nutrition framework must therefore consider not only an athlete’s genetic and microbial profile but also the specific physiological demands imposed by their sport and training regimen.

Endurance training is characterized by pronounced mitochondrial biogenesis and an increased reliance on oxidative phosphorylation, which in turn generates sustained oxidative stress. These adaptations suggest a heightened requirement for mitochondrial selenoproteins that preserve redox balance and mitochondrial integrity. Glutathione peroxidase 4 (GPx4) plays a unique and critical role in reducing lipid hydroperoxides within mitochondrial membranes, thereby protecting against ferroptosis and safeguarding electron transport chain function during prolonged exercise [43,44]. In endurance athletes, muscle GPx activity increases significantly in response to training, reflecting an adaptive mechanism to mitigate oxidative damage [45]. Selenoprotein T (SELENOT), another mitochondrial selenoprotein, contributes to the maintenance of ATP production and supports myoblast proliferation, linking it to both energy metabolism and muscle remodeling [72,73]. Selenoprotein O (SELENOO), although less extensively characterized, has also been implicated in mitochondrial redox homeostasis [74,75]. Collectively, these observations suggest that endurance athletes may derive particular benefit from nutritional strategies that support mitochondrial selenoprotein function, potentially enhancing oxidative capacity and delaying the onset of fatigue.

In contrast, strength and power adaptations are predominantly driven by myofibrillar protein turnover, calcium-mediated signaling, and satellite cell-mediated regeneration. These processes engage a distinct set of selenoproteins. Selenoprotein W (SELENOW) modulates protein turnover through the RAC1-mTOR axis, coordinating protein synthesis and degradation; its expression correlates positively with grip strength, and its loss exacerbates muscle wasting [65,69,73]. Selenoprotein N (SELENON) regulates calcium homeostasis within the endoplasmic reticulum by interacting with ryanodine receptors and the SERCA2 pump. Deficiencies in SELENON result in impaired excitation-contraction coupling and contribute to muscle fatigue, highlighting its importance in contractile function [69,70,71]. Selenoprotein K (SELENOK) is required for satellite cell-mediated myogenic differentiation and protects skeletal muscle from damage [66]. Additionally, several endoplasmic reticulum-resident selenoproteins, including SELENOF, SELENOS, and SELENOT (which also localizes to the ER), are involved in mitigating ER stress, a condition that may be exacerbated during periods of intense training [58,59]. For strength and power athletes, supporting these selenoproteins could optimize muscle remodeling, enhance contractile function, and accelerate recovery.

These observations lead to the hypothesis that selenium-biofortified probiotic interventions could be tailored to the specific demands of different sports. An endurance athlete with suboptimal functional selenium reserves might benefit from a formulation designed to support mitochondrial selenoproteins such as GPx4 and SELENOT, whereas a strength athlete with similar selenium insufficiency might require a regimen targeting SELENOW and SELENON. While grounded in mechanistic reasoning, this hypothesis requires direct testing in athletic populations. Future trials should therefore stratify participants not only by selenium status and genetic background but also by primary sport discipline, and should incorporate outcome measures that reflect the physiological demands of each discipline. For endurance athletes, relevant endpoints might include maximal oxygen consumption and time to exhaustion, whereas for strength athletes, measures such as one-repetition maximum and muscle cross-sectional area would be more appropriate. Such discipline-specific stratification will be essential for validating the concept of sport-tailored selenium interventions and for translating mechanistic insights into evidence-based practice.

**Figure 2:**
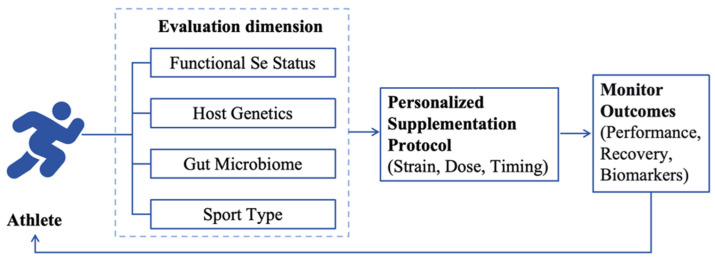
A proposed framework for stratified application of selenium-biofortified probiotics in precision sports nutrition. The flowchart illustrates a hypothesis-driven workflow for future clinical trials and personalized intervention design. Athletes are first assessed across four stratification dimensions: (1) functional selenium status using plasma SELENOP as a biomarker; (2) host genetic polymorphisms in selenoprotein-related genes (e.g., SEPS1, GPx); (3) baseline gut microbiome composition via metagenomic profiling; and (4) sport-specific physiological demands (endurance versus strength/power disciplines). These multidimensional data inform the design of personalized supplementation protocols, including selection of probiotic strain, selenium dosage, and intervention timing. Outcomes are monitored using relevant functional and performance measures, with iterative refinement of the protocol based on individual response. It must be emphasized that this framework remains conceptual and requires rigorous clinical validation through prospective, adequately powered trials before translation into practice.

Taken together, advancing selenium-biofortified probiotics necessitates a shift towards a precision nutrition model. This model employs SELENOP for initial functional screening, leverages multi-omics data for granular individual profiling, and tailors the nutritional priority based on the distinct selenoprotein demands imposed by different sports. Future research must focus on validating this stratified approach in athlete cohorts and developing practical clinical algorithms to integrate these complex data streams into actionable, personalized supplementation protocols. The stratification framework integrating functional selenium status, host genetics, microbiome composition, and sport-specific physiological demands provides a hypothesis-driven foundation for future investigations. It must be emphasized, however, that this framework remains conceptual and requires rigorous clinical validation before its components can be considered for translation into practice (Figure 2).

### 5.3. Advanced Delivery and Safety

As a live microbial product, selenium-biofortified probiotics present unique challenges for product standardization and quality control. Several critical parameters require strict specification to ensure reproducibility across studies and consistency in future commercial applications. These include probiotic strain identity and viability, total selenium content, selenium speciation profile (e.g., the proportion of selenomethionine, selenocysteine, and elemental selenium nanoparticles), and product stability under storage conditions as well as during transit through the gastrointestinal tract. Without standardization of these parameters, comparisons between studies will remain difficult, and the field will struggle to identify the most effective formulations. Establishing industry-wide consensus on these quality metrics should be a priority for future research collaborations.

Safety considerations are equally critical. Selenium has a narrow therapeutic window, and chronic overconsumption can lead to selenosis, characterized by symptoms ranging from gastrointestinal distress and hair loss to more severe neurological impairment. The European Food Safety Authority has established a tolerable upper intake level of 255 µg per day for adults, based on the prevention of selenosis in general populations [33]. For athletes, this value provides a useful safety benchmark, but its direct applicability is uncertain. Athletes may have higher selenium losses through sweat and urine during intense training, potentially increasing requirements, yet they may also be more susceptible to cumulative overload if supplementation is not carefully monitored. Sport-specific upper intake limits have not been established and warrant dedicated investigation.

Notably, the form of selenium delivered by probiotic biotransformation may influence its safety profile. Selenium nanoparticles synthesized by probiotic strains have demonstrated lower hepatic accumulation compared to inorganic selenium salts in animal models, while maintaining or even enhancing efficacy [23,105]. This “nanomodulation” approach may offer a favorable safety margin, though human data remain limited. Long-term safety studies in athletic populations, across different training loads and supplementation durations, are needed to establish evidence-based upper limits and to guide responsible use in practice.

The stratification framework proposed earlier, integrating functional selenium status, host genetics, microbiome composition, and sport-specific physiological demands, provides a hypothesis-driven foundation for future investigations. It must be emphasized, however, that this framework remains conceptual and requires rigorous clinical validation before its components can be considered for translation into practice. Prospective trials that incorporate product standardization, monitor safety outcomes, and test the efficacy of stratified approaches in adequately powered athlete cohorts will be essential for advancing selenium-biofortified probiotics from a promising concept to an evidence-based sports nutrition strategy.

## 6. Conclusions

Selenium-biofortified probiotics offer a promising synergistic nutritional strategy for mitigating exercise-induced oxidative stress, inflammation, gut barrier damage, and promoting recovery by integrating the antioxidant/anti-inflammatory properties of highly bioavailable organic selenium with the gut barrier-protective and immunomodulatory functions of probiotics, with the added potential to systemically support critical skeletal muscle selenoproteins involved in protein turnover, regeneration, calcium handling, and mitochondrial function. Strong evidence from animal experiments lays the foundation for its efficacy and preliminarily demonstrates advantages over traditional selenium supplementation. However, human application evidence remains insufficient and controversial, highlighting the importance of participant baseline status, supplementation protocol, and study design. Looking ahead, research in this field must progress towards deeper mechanistic dissection, particularly via multi-omics, rigorous and stratified clinical trials, and personalized application protocols based on selenium status and microbiome profiles. Through interdisciplinary collaboration, a thorough exploration of this “selenium–microbe” symbiont promises to catalyze the development of a new generation of microbiome and nutrigenomic-based precision sports nutrition solutions.

## Data Availability

No new data were created or analyzed in this study.
